# Prioritizing orphan proteins for further study using phylogenomics and gene expression profiles in *Streptomyces coelicolor*

**DOI:** 10.1186/1756-0500-4-325

**Published:** 2011-09-07

**Authors:** Mohammad Tauqeer Alam, Eriko Takano, Rainer Breitling

**Affiliations:** 1 Institute of Molecular, Cell and Systems Biology College of Medical, Veterinary and Life Sciences, Joseph Black Building B3.10, University of Glasgow, G12 8QQ, Glasgow, UK; 2 Groningen Bioinformatics Centre, Groningen Biomolecular Sciences and Biotechnology Institute, University of Groningen, The Netherlands; 3 Department of Microbial Physiology, Groningen Biomolecular Sciences and Biotechnology Institute, University of Groningen, The Netherlands

## Abstract

**Background:**

*Streptomyces coelicolor*, a model organism of antibiotic producing bacteria, has one of the largest genomes of the bacterial kingdom, including 7825 predicted protein coding genes. A large number of these genes, nearly 34%, are functionally orphan (hypothetical proteins with unknown function). However, in gene expression time course data, many of these functionally orphan genes show interesting expression patterns.

**Results:**

In this paper, we analyzed all functionally orphan genes of *Streptomyces coelicolor *and identified a list of "high priority" orphans by combining gene expression analysis and additional phylogenetic information (i.e. the level of evolutionary conservation of each protein).

**Conclusions:**

The prioritized orphan genes are promising candidates to be examined experimentally in the lab for further characterization of their function.

## Background

Here we present an analysis of orphan genes (hypothetical genes with unknown function) in the *Streptomyces coelicolor *genome, combining gene expression analysis and comparative genomics. The aim is to prioritize orphan genes for further study. In our gene expression studies [1,2], we frequently encountered genes that showed interesting expression patterns, but had no known function. To identify which of these genes merit in-depth experimental analysis, we developed a strategy for prioritizing protein encoding genes for additional characterization, combining phylogenomic information [3] (i.e. the level of evolutionary conservation of each protein), and gene expression data from a large gene expression time series [1]. We postulate that widely conserved proteins that show a physiologically relevant dynamic expression pattern are the most promising candidates for further experimental study, e.g. using gene overexpression and knock-out or knock-down approaches.

The functional annotation of orphan genes is not only relevant for its basic biological interest, but is also an important help for the improvement of genome-scale metabolic models based on genome annotation. These models in their initial form almost always contain gaps that need to be filled by manual curation or automated gap-filling algorithms that add missing essential metabolic activities to the models [2,4-7].

During our previous studies of genome-scale metabolic models of *Streptomyces coelicolor *and its relatives, we regularly had to postulate enzymatic functions that had not been assigned to specific proteins in the organisms [2,7]. Assigning specific enzyme-coding genes to these orphan metabolic activities is very important for the subsequent analysis and interpretation of the models, and several approaches have been developed to assign sequences to the orphan metabolic activities: they employ, for example, mRNA co-expression analysis [8], phylogenetic profile information [9-11], pattern recognition techniques [12] or comparative genomics [13]. These approaches are organism specific and have mostly been employed for well-studied model organisms such as *Escherichia coli *and *Saccharomyces cerevisiae*.

## Results and discussion

Of the 7825 predicted protein coding genes in the *Streptomyces coelicolor *genome [14], according to a re-annotation of the genome sequence in 2009, 2688 (34%) are coding for functionally orphan proteins, i.e. proteins that are annotated as "hypothetical protein", "conserved protein", "putative membrane protein" or "putative secreted protein". Of these orphan proteins, 26 are conserved in all and 381 are present in at least half (22/44) of the 44 analyzed complete actinomycete genomes (see Methods section for a complete species list). 683 orphan proteins are present in at least 11 (25%) and 177 are conserved in at least 33 (75%) actinomycete genomes.

Of the 381 generally conserved actinomycete orphan proteins (i.e., those that are present in at least half of the analyzed genomes), 25 are also encoded in all species in a selected set of diverse non-actinomycete bacterial genomes (*Bacillus subtilis, Escherichia coli K12, Lactobacillus plantarum WCFS1, Staphylococcus aureus*, and *Streptococcus pneumonia AP200*), and 73 are present in at least three of the representative bacterial genomes (see Additional File 1: Supplementary Table 1.xls).

Of these 73 ultra-highly conserved bacterial orphan genes, 22 also have putative homologues (reciprocal best BLAST hits) in at least half of the species in a representative set of eight non-bacterial genomes (the eukaryotes *Caenorhabditis elegans*, *Arabidopsis thaliana*, *Plasmodium falciparum*, *Drosophila melanogaster*, *Saccharomyces cerevisiae *and *Homo sapiens*, and the archaea *Haloterrigena turkmenica *and *Methanosarcina acetivorans*). These proteins are therefore almost universally conserved; however, although there seems to be significant conservation of some orphan proteins, none of them is truly universal, i.e. none has a putative homologue in all of the 58 studied genomes. This is most likely due to the fact that some of the included actinomycete genomes are highly reduced, as a result of the parasitic lifestyle of the organism, and the large phylogenetic distance covered (with the corresponding major differences in basic physiological processes).

To prioritize the orphan proteins for further characterization, we therefore summarized the phylogenomic information (i.e. the level of evolutionary conservation of each protein) in a single "conservation" score, which expresses the degree of conservation across the three domains examined (actinomycetes, non-actinomycete bacteria, non-bacteria). This score was combined with a second measure of expression dynamics across a large gene expression time series studying the metabolic switch caused by phosphate starvation. In this experiment, a fermenter culture of *S. coelicolor *was grown in phosphate-limited conditions, and gene expression data were obtained at 32 finely spaced time points throughout the duration of the experiment. Phosphate was depleted after about 35 hours, triggering a metabolic switch from primary to secondary metabolism, accompanied by a rapid global reorganization of the transcriptome, involving genes with a wide range of biological functions, from central metabolism and antibiotic biosynthesis, to cellular development and maintenance [1]. The "expression dynamics" score described in the Methods section identifies genes that show a smooth expression trend across (part of) the time series and favors those genes that show a particularly strong (step-like) expression change at one time point. This is intended to allow to focus on genes that are not only passively following the expression change during nutrient depletion but that show evidence for active regulation, which is indicative of a central function in cellular physiology. Based on the p-value of the "expression dynamics" score, we assigned a rank to each gene, and averaged this value with the rank of the "conservation" score. 734 orphan genes are significantly up- or down-regulated with expression dynamic p-values less than 0.00001 (significant after multiple-testing correction).

Using the averaged conservation and expression dynamics rank, we arrived at a list of 30 top orphan proteins. These were examined in more detail to determine if their function was really unknown: we checked the most recent versions of the Uniprot [15] and StrepDB database for annotations, performed a PSI-BLAST against the Uniprot database, compared the annotation of the homologs in *E. coli*, yeast and human where these were available, and analyzed the domain architecture using SMART tool (Simple Modular Architecture Research Tool) [16]. Using this information, we asked three microbiologist and bioinformaticians to independently score the genes according to their "orphanicity", i.e. their confidence in the absence of a known potential function. The three raters used a large collection of automatically provided evidence for all candidate genes, including annotation from the most recent versions of the Uniprot and StrepDB database, output of a PSI-BLAST against the Uniprot database, and the output of a domain architecture analysis using the SMART tool (Simple Modular Architecture Research Tool). In addition, they were free to do their own literature research and sequence analysis, although this did not generally identify useful extra information. The average score of the three raters was combined with the average score of the conservation and expression dynamics to arrive at a final ranking for the most interesting orphan genes for further study: the top genes are those for which we have absolutely no information about their function, that are ultra-highly conserved across species, and show a highly significant dynamics in their gene expression (Table 1).

**Table 1:** Top 30 orphan proteins for further study

Gene Name	Annotation	Final rank	Orphanicity rank	Exp. quantile	p-value	act	bac	non-bac
SCO1521	hypothetical protein	1	1	0.21	3.71E-10	44	5	5

SCO2301	hypothetical protein	6	4	0.34	3.27E-07	43	5	5

SCO5362	hypothetical protein	6.5	9	0.13	2.02E-07	44	4	7

SCO1769	hypothetical protein	8	5	0.12	3.08E-08	40	3	1

SCO5746	hypothetical protein	8	7	0.18	4.38E-18	20	3	1

SCO3882	hypothetical protein	8.5	2	0.18	6.71E-08	38	5	1

SCO5546	hypothetical protein	8.5	14	0.35	7.62E-09	42	3	6

SCO5745	hypothetical protein	9.5	17	0.02	9.49E-10	43	4	6

SCO1925	hypothetical protein	11.5	18	0.09	1.24E-07	44	5	3

SCO2577	hypothetical protein	12	3	0.64	2.66E-07	41	5	1

SCO1676	hypothetical protein	12.5	15	0.32	7.05E-09	31	1	4

SCO1919	hypothetical protein	12.5	11	0.16	5.74E-07	44	4	2

SCO5491	hypothetical protein	12.5	6	0.35	3.07E-07	32	3	3

SCO2081	hypothetical protein	13	8	0.60	2.88E-08	38	2	1

SCO2902	hypothetical protein	14.5	22	0.37	3.05E-07	43	5	4

SCO1522	hypothetical protein	15.5	19	0.19	6.47E-07	43	3	5

SCO1920	hypothetical protein	16	12	0.27	1.71E-06	42	5	5

SCO3839	hypothetical protein	16.5	27	0.35	1.60E-08	35	3	2

SCO3960	hypothetical protein	17.5	13	0.30	5.66E-08	29	5	1

SCO2901	hypothetical protein	18	23	0.36	5.37E-07	41	3	5

SCO1924	hypothetical protein	18.5	20	0.08	6.81E-08	44	1	2

SCO6766	hypothetical protein	18.5	10	0.19	4.55E-08	20	1	2

SCO1775	hypothetical protein	21	16	0.32	3.00E-06	42	4	2

SCO1222	hypothetical protein	22	21	0.43	3.33E-09	27	1	1

SCO5645	hypothetical protein	22	28	0.07	3.11E-07	36	4	2

SCO1530	hypothetical protein	24.5	24	0.03	8.99E-07	43	1	5

SCO2497	hypothetical protein	26.5	29	0.52	2.38E-06	37	5	7

SCO5787	hypothetical protein	27	26	0.12	5.88E-06	44	3	7

SCO2599	hypothetical protein	27.5	25	0.13	4.17E-07	44	1	1

SCO5711	hypothetical protein	29.5	30	0.12	8.65E-06	44	5	5

The proteins are prioritized according to their conservation across actinomycetes, bacteria and non-bacteria; their expression dynamics (summarized in the p-value); and their orphanicity, i.e. the absence of any functional information, assessed as described in the text.

**Figure 1:**
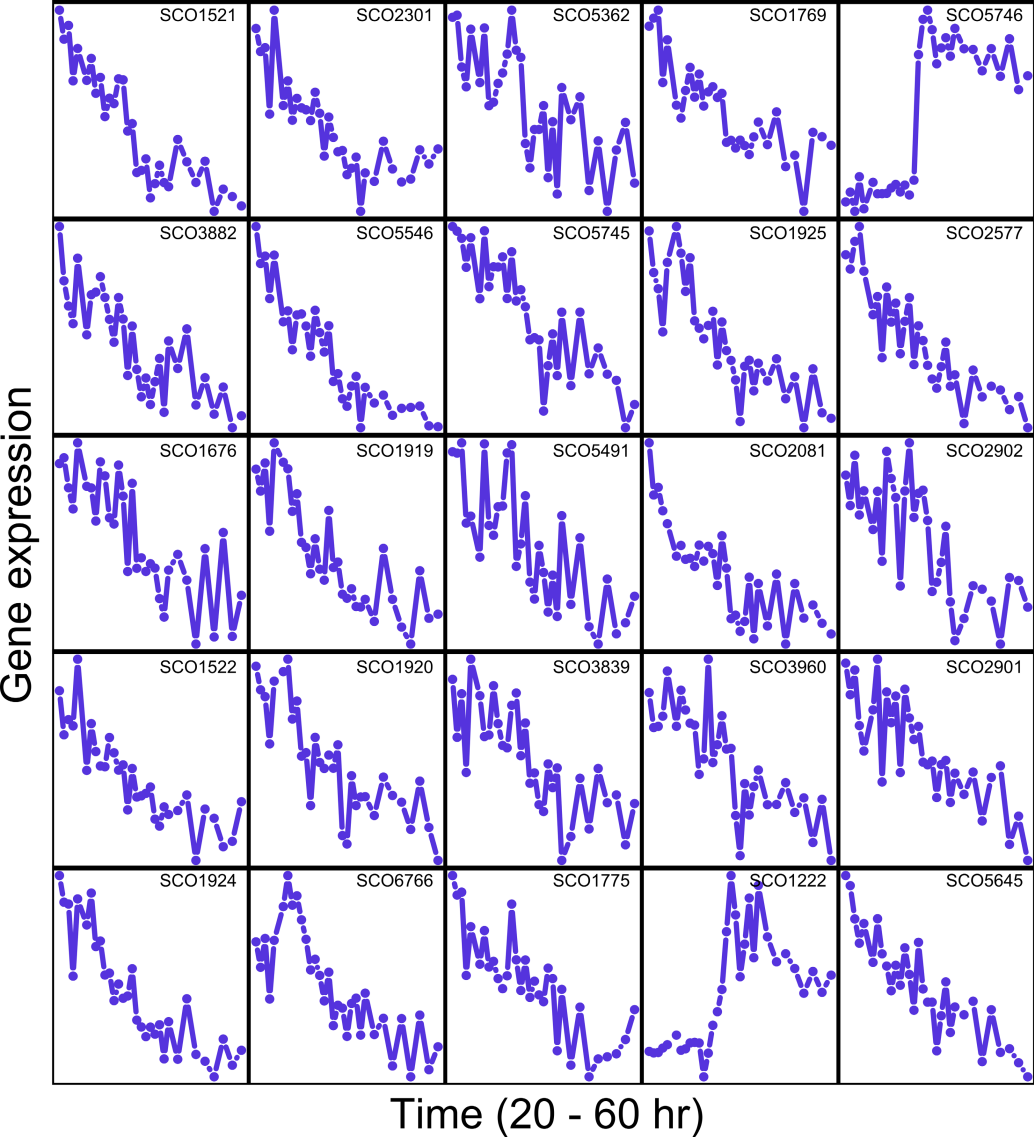
**Average expression profile of the top 25 candidate orphan genes**. This figure shows the expression profiles of the candidate genes during the phosphate-starvation experiment described in the text. Phosphate depletion occurs between time point 15 and 16 (i.e., between 35 and 36 hours after the start of the culture).

Based on the gene expression profiles (Figure 1), the candidate genes SCO5746 and SCO1222 are particularly interesting: they show a very strong switch upon phosphate starvation, and their expression increases in stationary phase similar to the expression pattern of the antibiotic biosynthesis gene clusters *act *and *red*. All other genes show a decrease of expression along the time course. SCO5746 has a putative uncharacterized homolog in *E. coli *and contains a domain of the DegT/DnrJ/EryC1/StrS aminotransferase family. The aminotransferase activity was demonstrated for purified StsC protein, which acts as an L-glutamine:scyllo-inosose aminotransferase and catalyses the first amino acid transfer in the biosynthesis of the streptidine subunit of antibiotic streptomycin. It is therefore tempting to speculate that the SCO5746 gene has some role in the biosynthesis of a new antibiotic in *S. coelicolor *as well, and the same might be the case for the completely uncharacterized SCO1222. The closest putative antibiotic biosynthesis clusters are SCO5799-SCO5801 (siderophore synthetase type) and SCO1206-SCO1208 (chalcone synthetase type; [17]), both of which seem unlikely candidates for interacting with SCO5746 or SCO1222. However, it is possible that these genes contribute to a dispersed biosynthetic pathway, not involving a dense genomic clustering. Of course, they could also be contributing to any other stationary phase process.

**Figure 2:**
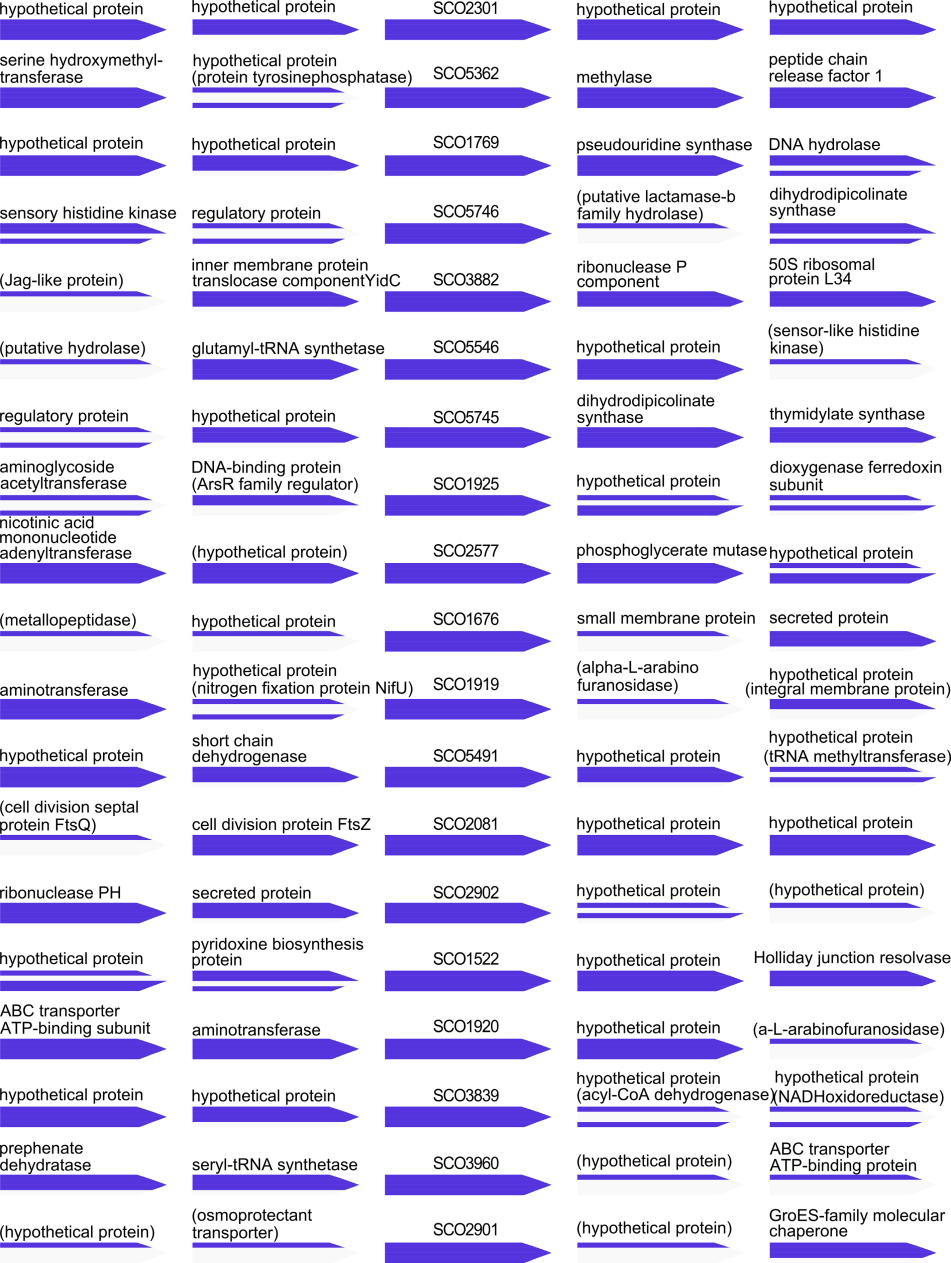
**Neighborhood conservation of the top 20 candidate orphan genes**. This figure shows annotation conservation of the neighbors of orphan genes in four sequenced *Streptomyces *genomes. The conserved orphan gene is shown in the centre, and the two neighbors on each side are shown in the form of arrows. Each arrow has four sections, corresponding to the four *Streptomyces *species: *S. coelicolor, S. avermitilis, S. griseus and S. scabies*. They are colored in blue where the annotation matches that of *S. coelicolor*. The annotation of the *S. coelicolor *homolog is listed above each gene if it is conserved in at least one of the other species; if at least two of the other species share another annotation, this is listed in brackets.

Interestingly, we see a strong neighborhood conservation of most of the candidate orphan genes in other *Streptomyces *species (Figure 2). In some cases, the annotation of the neighbors does suggest at least a broad functional category: for example, SCO1521/1522 might be involved in DNA remodeling during recombination, as their conserved neighbors are a Holliday junction resolvase and DNA helicase (RuvABC complex); and SCO2081 might play a role in cell division, matching its conserved neighbor, the cell division protein ftsZ [18]. However, most of the conserved neighbors are hypothetical proteins themselves and do not seem to immediately identify a putative function for most of the orphan genes; nonetheless, the neighborhood information will be valuable for the design and interpretation of the most efficient experimental perturbations. The dynamic expression pattern of each of the neighborhoods depicted in Figure 2 is shown in Additional File 2: SupplementaryFile1.pdf. This illustrates, e.g., that the expression of SCO1522 shows a very similar expression trend compared with its left and right neighbors (SCO1521 and SCO1523), confirming the relevance of adjacency on the genome for predicting gene functionality.

The prioritization reported in this paper of course depends on implicit assumptions about what constitutes a protein of interest. Here we were *a priori *interested in any protein that is maintained by purifying selection in a large number of genomes, indicating that it is involved in a generally important physiological process. On the other hand, we assumed that genes that show strong gene expression responses to a major physiological perturbation are more likely to be functionally relevant under the conditions studied. Genes that are not expressed or not finely controlled are more likely to have more specialized functions. This approach does not exclude the identification of housekeeping genes, which may not be directly involved in the physiological process studied in the gene expression analysis, as these genes still tend to show dynamic expression patterns (as evidenced, e.g., by the ribosomal protein genes [1]). The results are, however, affected by the availability of gene expression data sets and will become more informative once other large-scale expression studies, comparable to the one used here, become available.

## Conclusions

Our aim was to prioritize protein coding orphan genes (hypothetical proteins with unknown function) for further experimental characterization of their function. We developed an algorithm to detect dynamic switches in a large gene expression time course data set, and assigned an "expression dynamics" score to every orphan gene, arguing that genes that show substantial expression changes corresponding to biologically relevant events would be most interesting to follow up. We also summarized the available evolutionary information in a "conservation" score across a broad range of organisms (many actinomycetes, other bacteria and various non-bacterial species). We combined the "expression dynamics" rank and "conservation" rank to identify a robust list of 30 high priority orphan genes, which are promising candidates for detailed experimental study.

## Methods

### Genome sequence analysis

For the phylogenomic profiling, we studied the complete genome sequences of the 44 actinomycete species, which were also used in our earlier phylogenetic study [3]: *Arthrobacter aurescens *TC1, *Acidothermus cellulolyticus *11B, *Bifidobacterium adolescentis *ATCC 15703, *Bifidobacterium longum *NCC2705, *Corynebacterium diphtheriae *NCTC 13129, *Corynebacterium efficiens *YS-314, *Corynebacterium glutamicum *ATCC 13032, *Corynebacterium jeikeium *K411, *Clavibacter michiganensis subsp michiganensis *NCPPB 382, *Frankia alni *ACN14a, *Frankia sp *CcI3, *Frankia sp *EAN1pec, *Kineococcus radiotolerans *SRS30216, *Leifsonia xyli *subsp xyli str CTCB07, *Mycobacterium avium *subsp, paratuberculosis str k10, *Mycobacterium avium *104, *Mycobacterium bovis *BCG Pasteur 1173P2, *Mycobacterium bovis *subsp bovis AF2122 97, *Mycobacterium gilvum *PYR-GCK, *Mycobacterium sp *JLS, *Mycobacterium sp *KMS, *Mycobacterium leprae *TN, *Mycobacterium sp *MCS, *Mycobacterium tuberculosis *H37Ra, *Mycobacterium smegmatis *str MC2155, *Mycobacterium tuberculosis *CDC1551, *Mycobacterium tuberculosis *F11, *Mycobacterium tuberculosis *H37Rv, *Mycobacterium ulcerans *Agy99, *Mycobacterium vanbaalenii *PYR-1, *Nocardioides sp *JS614, *Nocardia farcinica *IFM 10152, *Propionibacterium acnes *KPA171202, *Rhodococcus sp *RHA1, *Renibacterium salmoninarum *ATCC 33209, *Salinispora arenicola *CNS 205, *Streptomyces avermitilis *MA 4680, *Saccharopolyspora erythraea *NRRL 2338, *Streptomyces griseus *strain IFO13350, *Streptomyces scabies *strain 8722, *Salinispora tropica *CNB 440, *Thermobifida fusca *YX, *Tropheryma whipplei *str Twist, *Tropheryma whipplei *TW08 27. This was complemented by the genomes of 6 eukaryotes (*Caenorhabditis elegans*, *Arabidopsis thaliana*, *Homo sapiens*, *Plasmodium falciparum *3D7, *Drosophila melanogaster*, *Saccharomyces cerevisiae*), 2 archaea (*Haloterrigena turkmenica*, *Methanosarcina acetivorans*), and 5 other model bacteria from different taxonomical classes (*Bacillus subtilis*, *Escherichia coli K12*, *Lactobacillus plantarum *WCFS1, *Staphylococcus aureus*, *Streptococcus pneumonia *AP200). Putative homologs were identified as reciprocal best BLAST hits. The conservation score was calculated in three steps: (1) the genes were independently ranked according to the number of species of actinomycetes, other bacteria, and non-bacteria in which they have a putative homolog; (2) their ranks in the bacteria and non-bacteria lists were averaged; and (3) the resulting rank and the rank in the actinomycete list were averaged again to produce the final rank.

### Gene expression data

Details about the gene expression dataset and experimental conditions can be found in [1,2]. Briefly, mRNA abundance was assessed at 32 time points during a 68-hour phosphate-limited fermentor culture of *S. coelicolor*, using custom-designed Affymetrix genechips; the data reveal a complex sequence of gene expression switches, affecting a large diversity of biological processes, from phosphate uptake to secondary metabolism and protein biosynthesis.

### Dynamic expression detection

To identify genes that show a dynamic expression along the time course, and in particular genes that have a clear expression switch at one time point, we used the following iterative algorithm (in pseudo code):

Input: a vector *v *of gene expression data

Output: minPvalue, the p-value of the switch-like dynamic expression

Initialize: minPvalue: = 1

For each value *i *in the set (2 to (length(*v*) - 2)), do

*j *: = *i *+ 1

MaxWindowSize < - min(*i*, length (*v*) - *i*)

For each position *p *in the set ((*i *- MaxWindowSize + 1) to *i *- 1), do

*q *: = *j *+ (*i *- *p*)

Pvalue: = p-value of the t-test comparing *v*[*p:i*] and *v*[*j:q*]

If (Pvalue < minPvalue), then

minPvalue: = Pvalue

end

end

end

return minPvalue

An R implementation of the algorithm is available from the authors upon request.

## Competing interests

The authors declare that they have no competing interests.

## Authors' contributions

ET and RB designed the study. MTA performed the analysis. RB and MTA wrote the manuscript. All authors revised the final manuscript.

## Supplementary Material

Additional file 1 **Table of generally conserved actinomycete orphan proteins**. The table lists 381 *Streptomyces coelicolor *orphan genes that are generally conserved in other actinomycetes genomes (more than half of the 44 actinomycete species examined; blue gene numbers). Of these, the top 73 are present in at least three of five representative non-actinomycete bacterial genomes (red; 25 are present in all five of these species). The top 22 genes also have putative homologues (reciprocal best BLAST hits) in at least half of the species in a representative set of eight non-bacterial genomes (green). Click here for file

Additional file 2 **Gene expression profile of gene neighborhoods**. Expression profile of the genes shown in Figure 2. Click here for file

## References

[B1] Nieselt K Battke F Herbig A Bruheim P Wentzel A Jakobsen ØM Sletta H Alam MT Merlo ME Moore J Omara WAM Morrissey ER Juarez-Hermosillo MA Rodríguez-García A Nentwich M Thomas L Iqbal M Legaie R Gaze WH Challis GL Jansen RC Dijkhuizen L Rand DA Wild DL Bonin M Reuther J Wohlleben W Smith MCM Burroughs NJ Martín JF Hodgson DA Takano E Breitling R Ellingsen TE Wellington EMH The dynamic architecture of the metabolic switch in *Streptomyces coelicolor* BMC Genomics 2010 11 10 10.1186/1471-2164-11-10 20053288 PMC2824715

[B2] Alam MT Merlo ME Hodgson DA Wellington EMH Takano E Breitling R Metabolic modeling and analysis of the metabolic switch in *Streptomyces coelicolor* BMC Genomics 2010 11 202 10.1186/1471-2164-11-202 20338070 PMC2853524

[B3] Alam MT Merlo ME Takano E Breitling R Genome-based phylogenetic analysis of *Streptomyces *and its relatives Mol Phylogenet Evol 2010 54 763 772 10.1016/j.ympev.2009.11.019 19948233

[B4] Thiele I Palsson BO A protocol for generating a high-quality genome-scale metabolic reconstruction Nat Protocols 2010 5 93 121 10.1038/nprot.2009.203 PMC3125167 20057383

[B5] Henry CS DeJongh M Best AA Frybarger PM Linsay B Stevens RL High-throughput generation, optimization and analysis of genome-scale metabolic models Nat Biotech 2010 28 977 982 10.1038/nbt.1672 20802497

[B6] Satish Kumar V Dasika M Maranas C Optimization based automated curation of metabolic reconstructions BMC Bioinformatics 2007 8 212 10.1186/1471-2105-8-212 17584497 PMC1933441

[B7] Medema MH Trefzer A Kovalchuk A van den Berg M Müller U Heijne W Wu L Alam MT Ronning CM Nierman WC Bovenberg RAL Breitling R Takano E The Sequence of a 1.8-Mb Bacterial Linear Plasmid Reveals a Rich Evolutionary Reservoir of Secondary Metabolic Pathways Genome Biology and Evolution 2010 2 212 224 10.1093/gbe/evq013 20624727 PMC2997539

[B8] Kharchenko P Vitkup D Church GM Filling gaps in a metabolic network using expression information Bioinformatics 2004 20 Suppl 1 i178 185 10.1093/bioinformatics/bth930 15262797

[B9] Jothi R Przytycka TM Aravind L Discovering functional linkages and uncharacterized cellular pathways using phylogenetic profile comparisons: a comprehensive assessment BMC Bioinformatics 2007 8 173 10.1186/1471-2105-8-173 17521444 PMC1904249

[B10] Chen L Vitkup D Predicting genes for orphan metabolic activities using phylogenetic profiles Genome Biol 2006 7 R17 10.1186/gb-2006-7-2-r17 16507154 PMC1431735

[B11] Snitkin ES Gustafson AM Mellor J Wu J DeLisi C Comparative assessment of performance and genome dependence among phylogenetic profiling methods BMC Bioinformatics 2006 7 420 10.1186/1471-2105-7-420 17005048 PMC1592128

[B12] Cuff AL Sillitoe I Lewis T Redfern OC Garratt R Thornton J Orengo CA The CATH classification revisited--architectures reviewed and new ways to characterize structural divergence in superfamilies Nucleic Acids Res 2009 37 D310 314 10.1093/nar/gkn877 18996897 PMC2686597

[B13] Osterman A Overbeek R Missing genes in metabolic pathways: a comparative genomics approach Current Opinion in Chemical Biology 2003 7 238 251 10.1016/S1367-5931(03)00027-9 12714058

[B14] Bentley SD Chater KF Cerdeño-Tárraga A-M Challis GL Thomson NR James KD Harris DE Quail MA Kieser H Harper D Bateman A Brown S Chandra G Chen CW Collins M Cronin A Fraser A Goble A Hidalgo J Hornsby T Howarth S Huang C-H Kieser T Larke L Murphy L Oliver K O'Neil S Rabbinowitsch E Rajandream M-A Rutherford K Rutter S Seeger K Saunders D Sharp S Squares R Squares S Taylor K Warren T Wietzorrek A Woodward J Barrell BG Parkhill J Hopwood DA Complete genome sequence of the model actinomycete *Streptomyces coelicolor *A3(2) Nature 2002 417 141 147 10.1038/417141a 12000953

[B15] The UniProt Consortium The Universal Protein Resource (UniProt) in 2010 Nucleic Acids Research 2009 38 D142 D148 19843607 10.1093/nar/gkp846 PMC2808944

[B16] Schultz J Milpetz F Bork P Ponting CP SMART, a simple modular architecture research tool: Identification of signaling domains Proceedings of the National Academy of Sciences of the United States of America 1998 95 5857 5864 10.1073/pnas.95.11.5857 9600884 PMC34487

[B17] Zhao B Guengerich FP Bellamine A Lamb DC Izumikawa M Lei L Podust LM Sundaramoorthy M Kalaitzis JA Reddy LM Kelly SL Moore BS Stec D Voehler M Falck JR Shimada T Waterman MR Binding of two flaviolin substrate molecules, oxidative coupling, and crystal structure of *Streptomyces coelicolor *A3(2) cytochrome P450 158A2 J Biol Chem 2005 280 11599 11607 10.1074/jbc.M410933200 15659395

[B18] Jakimowicz D Gust B Zakrzewska-Czerwinska J Chater KF Developmental-Stage-Specific Assembly of ParB Complexes in *Streptomyces coelicolor *Hyphae J Bacteriol 2005 187 3572 3580 10.1128/JB.187.10.3572-3580.2005 15866947 PMC1112017

