# Supplementary material for: Prioritizing orphan proteins for further study using phylogenomics and gene expression profiles in Streptomyces coelicolor
Source: BMC Res Notes. 2011 Sep 7;4:325. doi: 10.1186/1756-0500-4-325 (PMC3224560; doi:10.1186/1756-0500-4-325)
Supplement: Additional file 2 — Gene expression profile of gene neighborhoods. Expression profile of the genes shown in Figure 2. [file 1756-0500-4-325-S2.PDF]

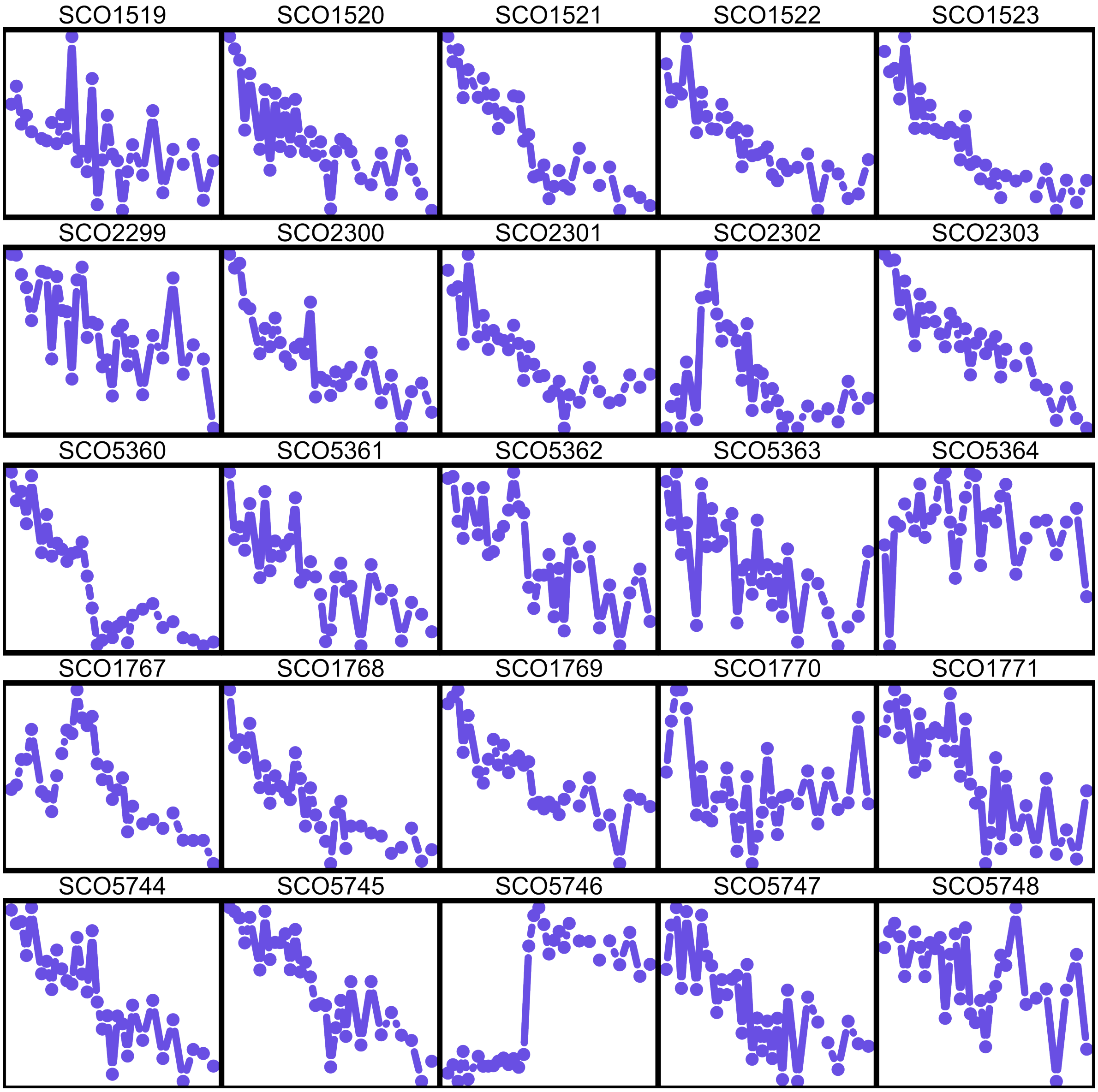
SCO1519
SCO1520
SCO1521
SCO1522
SCO1523
SCO2299
SCO2300
SCO2301
SCO2302
SCO2303
SCO5360
SCO5361
SCO5362
SCO5363
SCO5364
SCO1767
SCO1768
SCO1769
SCO1770
SCO1771
SCO5744
SCO5745
SCO5746
SCO5747
SCO5748

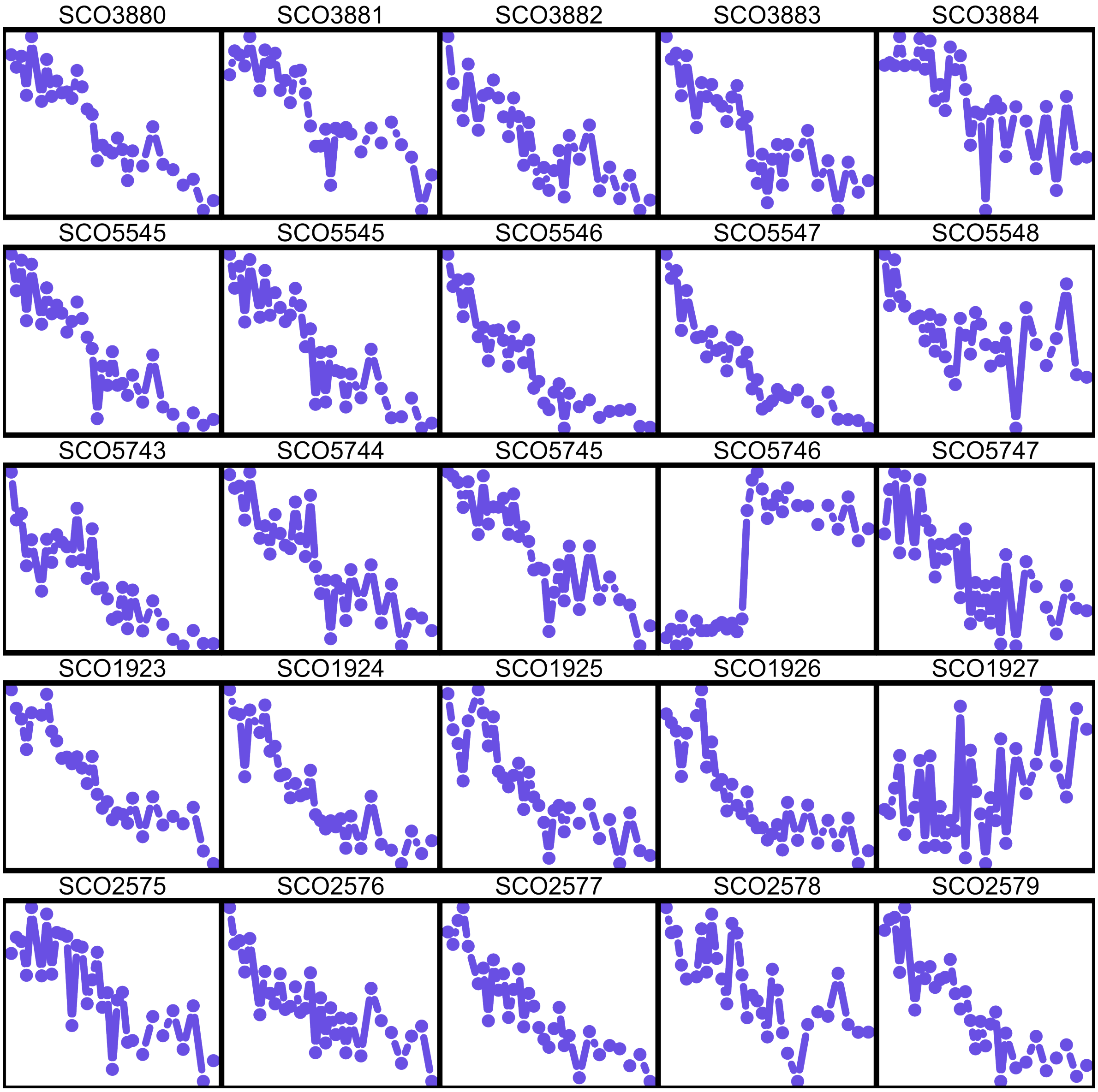

SCO3880
SCO3881
SCO3882
SCO3883
SCO3884
SCO5545
SCO5545
SCO5546
SCO5547
SCO5548
SCO5743
SCO5744
SCO5745
SCO5746
SCO5747
SCO1923
SCO1924
SCO1925
SCO1926
SCO1927
SCO2575
SCO2576
SCO2577
SCO2578
SCO2579

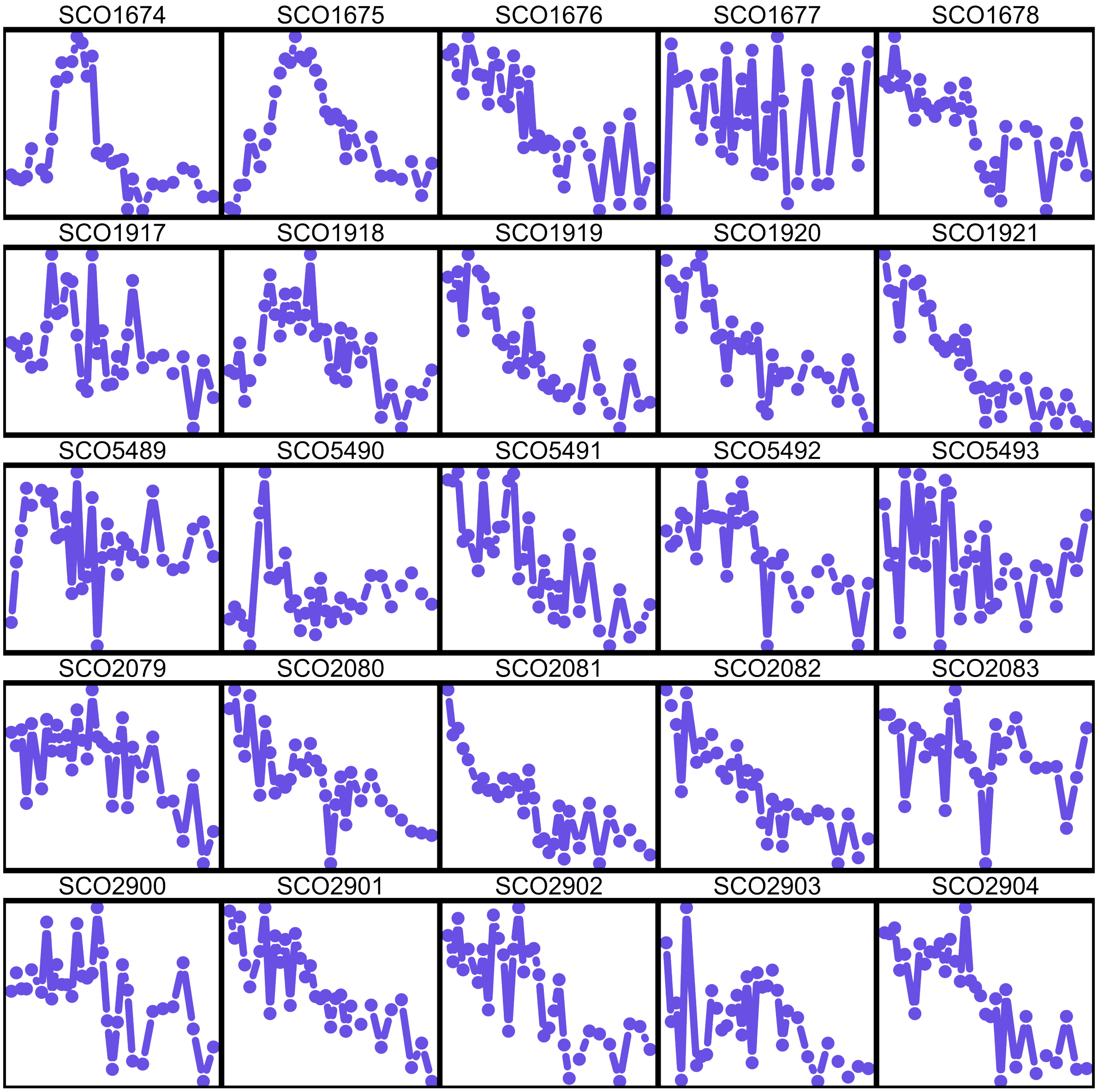
SCO1674
SCO1675
SCO1676
SCO1677
SCO1678
SCO1917
SCO1918
SCO1919
SCO1920
SCO1921
SCO5489
SCO5490
SCO5491
SCO5492
SCO5493
SCO2079
SCO2080
SCO2081
SCO2082
SCO2083
SCO2900
SCO2901
SCO2902
SCO2903
SCO2904

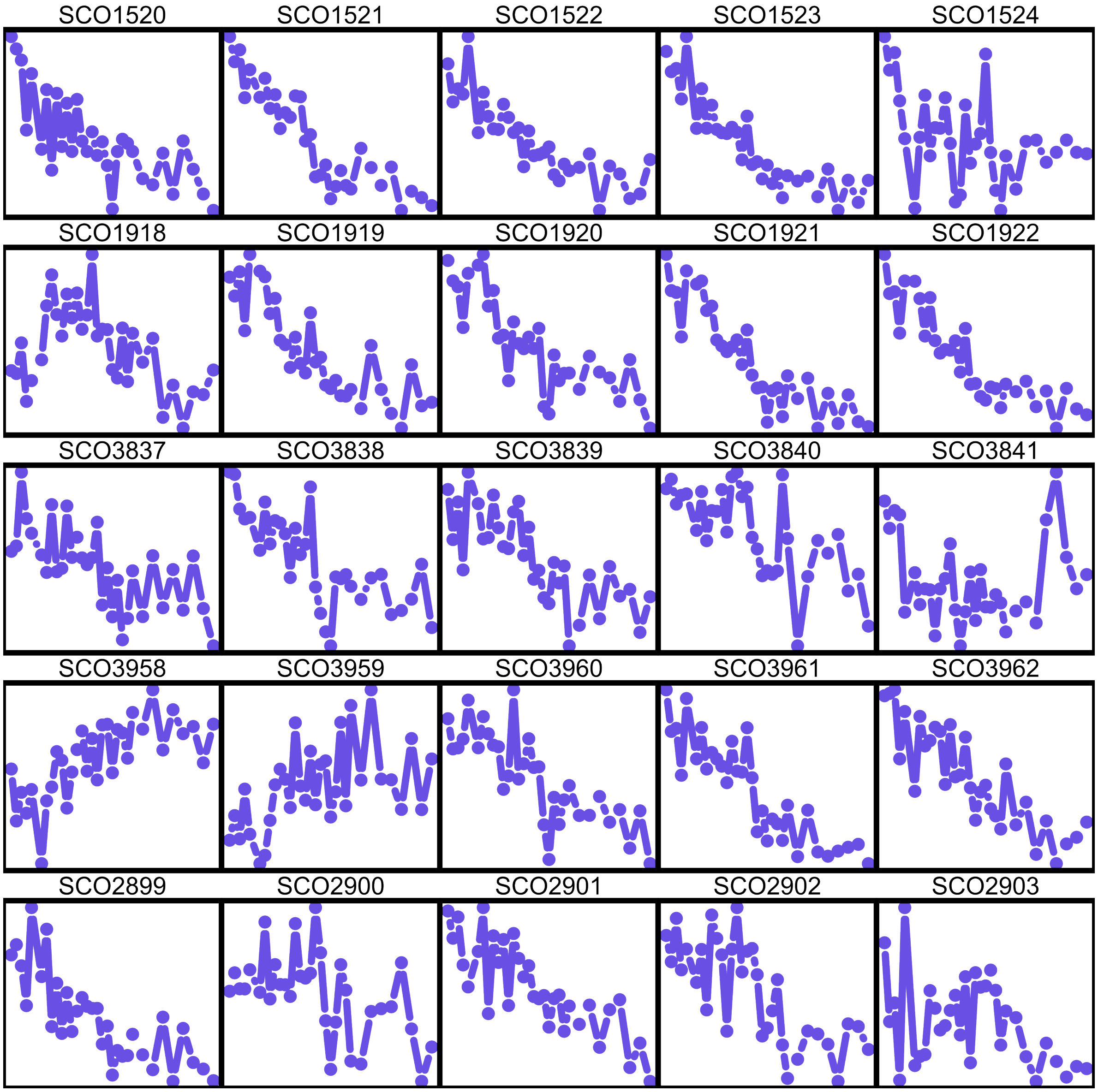

The dynamic expression pattern of each of the neighborhoods of top 20 orphan genes depicted in Figure 1.
